# Identification of an Optimal COVID-19 Booster Allocation Strategy to Minimize Hospital Bed-Days with a Fixed Healthcare Budget

**DOI:** 10.3390/vaccines11020377

**Published:** 2023-02-07

**Authors:** Ritika Kapoor, Baudouin Standaert, Edmund J. Pezalla, Nadia Demarteau, Kelly Sutton, Eszter Tichy, George Bungey, Sofie Arnetorp, Klas Bergenheim, Duncan Darroch-Thompson, Wilhelmine Meeraus, Lucas M. Okumura, Renata Tiene de Carvalho Yokota, Ray Gani, Terry Nolan

**Affiliations:** 1 Evidera, PPD Singapore, 08–11, 1 Fusionopolis Walk, Singapore 138628, Singapore; 2 Faculty of Medicine and Life Sciences, University of Hasselt, Agoralaan, 3590 Diepenbeek, Belgium; 3 Enlightenment Bioconsult, LLC, 140 S Beach Street, Suite 310, Daytona Beach, FL 32114, USA; 4 Evidera, 1932 Brussels, Belgium; 5 Evidera, Melbourne, VIC 3004, Australia; 6 Evidera, H-1113 Budapest, Hungary; 7 Evidera, PPD the Ark, 2nd Floor, 201 Talgarth Road, London W6 8BJ, UK; 8 Health Economics & Payer Evidence, BioPharmaceuticals R&D, AstraZeneca, 431 83 Gothenberg, Sweden; 9 International Market Access, Vaccines and Immune Therapies, AstraZeneca, Singapore 339510, Singapore; 10 Medical Evidence, Vaccines and Immune Therapies, AstraZeneca, Cambridge CB2 8PA, UK; 11 Health Economics & Payer Evidence, BioPharmaceuticals R&D, AstraZeneca, São Paulo 06709-000, Brazil; 12 P95 Epidemiology & Pharmacovigilance, 3001 Leuven, Belgium; 13 The Peter Doherty Institute for Infection and Immunity, University of Melbourne, Parkville, VIC 3010, Australia; 14 Murdoch Children’s Research Institute, Parkville, VIC 3052, Australia

**Keywords:** COVID-19 vaccination, booster, constrained optimization model, budget constraint, booster allocation, budget and healthcare resources

## Abstract

Healthcare decision-makers face difficult decisions regarding COVID-19 booster selection given limited budgets and the need to maximize healthcare gain. A constrained optimization (CO) model was developed to identify booster allocation strategies that minimize bed-days by varying the proportion of the eligible population receiving different boosters, stratified by age, and given limited healthcare expenditure. Three booster options were included: B_1_, costing US $1 per dose, B_2_, costing US $2, and no booster (NB), costing US $0. B_1_ and B_2_ were assumed to be 55%/75% effective against mild/moderate COVID-19, respectively, and 90% effective against severe/critical COVID-19. Healthcare expenditure was limited to US$2.10 per person; the minimum expected expense using B_1,_ B_2,_ or NB for all. Brazil was the base-case country. The model demonstrated that B_1_ for those aged <70 years and B_2_ for those ≥70 years were optimal for minimizing bed-days. Compared with NB, bed-days were reduced by 75%, hospital admissions by 68%, and intensive care unit admissions by 90%. Total costs were reduced by 60% with medical resource use reduced by 81%. This illustrates that the CO model can be used by healthcare decision-makers to implement vaccine booster allocation strategies that provide the best healthcare outcomes in a broad range of contexts.

## 1. Introduction

The COVID-19 pandemic is in its third year and continues to seriously impact global public health [1,2], with almost 600 million confirmed cases and nearly 6.5 million deaths reported worldwide as of August 2022 [3]. While non-pharmaceutical interventions such as strict lockdowns, mask wearing, and restricting social and economic activities have helped limit the spread of the SARS-CoV-2 virus [4], vaccination is widely considered the most efficient strategy for preventing severe disease, increasing population immunity, and reducing the ongoing COVID-19 health crisis [5,6].

Despite widespread implementation of COVID-19 vaccination, waning vaccine effectiveness and the emergence of novel SARS-CoV-2 variants have resulted in frequent local epidemics and breakthrough infections [7,8,9,10]. Booster vaccinations (including recently developed multivalent vaccines) increase the levels of antibodies against both wild-type and variant SARS-CoV-2, counteracting the waning of SARS-CoV-2 immunogenicity and providing a wider breadth of immunity against variants of concern [11,12,13,14]. Recent clinical data demonstrate that a COVID-19 booster dose given after the primary vaccine series has high effectiveness against infection [2,15,16], symptomatic disease [17], mortality [16], and hospitalizations or emergency care encounters [15,18,19], even during periods when the Delta or Omicron variants were dominant. As a result, the World Health Organization (WHO) recommends booster vaccination for the general population and additional booster doses for people who are immunocompromised [20].

Although boosters help reduce both the incidence and severity of COVID-19 and the resulting burden on healthcare systems [21], nationwide booster programs may significantly impact healthcare budgets [22]. To further complicate decision making, many different booster options are available, with varying effectiveness profiles and purchase prices [23]. Public health payers must decide which booster option to use to maximize healthcare benefits, while ensuring that total system costs remain affordable [3].

Constrained optimization (CO) methods identify the best solution to a problem to obtain the optimal result while complying with all relevant constraints, such as financial, logistical, or human resource limitations [24]. In the healthcare arena, CO can help decision makers to identify and prioritize those treatment strategies and interventions that will maximize health gains when faced with budget constraints, and is therefore a useful tool for aiding economically efficient decisions [24]. CO models have been used to optimize vaccination programs for various infectious diseases [25,26,27,28], identify optimal cancer prevention strategies [29,30], and prioritize COVID-19 vaccine allocation [28,31,32,33,34,35,36,37].

This article describes a CO model developed to identify the best COVID-19 booster allocation strategies (BAS) for age-identified subpopulations under different conditions. The aim is to provide a flexible tool to help healthcare decision makers to identify the best combinations of COVID-19 boosters in line with their budget, so that health outcomes can be maximized when healthcare resources are limited. The CO model was developed from the perspective of local health authorities in Brazil. Brazil was chosen as the base case country as it was one of the most highly impacted countries in terms of COVID-19 incidence, resulting in healthcare budget restrictions on vaccination programs [38,39,40,41,42]. However, the model can be easily adapted to any country to guide optimal booster dose allocation within local budgetary constraints.

## 2. Materials and Methods

### 2.1. Constrained Optimization Model

This CO model is a formal approach to distributing a fixed sum (vaccination budget) in a way that minimizes the hospital-bed days resulting from SARS-CoV-2 infection. It is a method of optimizing a specific outcome measure by determining the best combination of decision variables expressed in a mathematical equation called the objective function [43]. Our CO model was developed in Microsoft (MS) Excel to identify the best BAS that would achieve the objective of minimizing COVID-19 hospital bed-days, given a predefined healthcare budget (see Constrained Optimization Mathematical Model in Supplementary Material). For the analysis, two hypothetical boosters, B_1_ and B_2_, were considered after primary immunization with two doses of any COVID-19 vaccine. B_1_ and B_2_ were both assumed to be 90% effective at preventing severe (hospitalization requiring admission to an intensive care unit [ICU] without mechanical ventilation [MV]) and critical (hospitalization requiring MV) COVID-19, but they had different effectiveness at preventing mild (symptomatic, no hospitalization) and moderate (hospitalization requiring admission to a general ward only) COVID-19 (55% for B_1_ and 75% for B_2_) [44]. The study took a conservative approach in considering a time horizon of 3 months and including the average effectiveness for each booster in the first 3 months after the booster [44]. Owing to the unavailability of data at the time of model creation, effectiveness was assumed to be independent of which primary vaccine was given. Each booster was assumed to have a different cost per dose, US $1 (all dollar amounts are in US $) for B_1_ and US $2 for B_2_.

The CO model was designed to identify a BAS that could include any combination of the different boosters or no booster (NB). Only adults were considered in the analysis, allocated to the following age-groups: 18–29, 30–39, 40–49, 50–59, 60–69, 70–79, and ≥80 years. These age groups were chosen to enable different booster allocation for different age bands, so that an optimal BAS could be identified. Adults who had completed their primary vaccination 6 months prior to booster administration were included as the target population, because the booster effectiveness values were based on an adult population. The model accounted for the prevalence of co-morbidities and their impact on the risk of developing severe COVID-19 at each age stratification. A decision tree model was developed to calculate the cost and health outcomes of implementing the different booster options. Using these outcomes, the CO model adjusted the allocation to B_1_, B_2_, or NB with the MS Excel add-in Solver software for the target population by age-group.

A maximum budget of $2.10 per person was selected in the base case, which closely represents the lowest expected per capita cost of any of the booster options (B_1_, B_2_, or NB) when implemented for the entire adult population (the lowest expected cost is $2.04 per person for the B_1_ strategy for all). This budget represented the total costs inclusive of the booster costs and medical resource use (MRU) costs for COVID-19 treatment in case of infection with SARS-CoV-2.

### 2.2. Decision Tree Model

**Figure 1:**
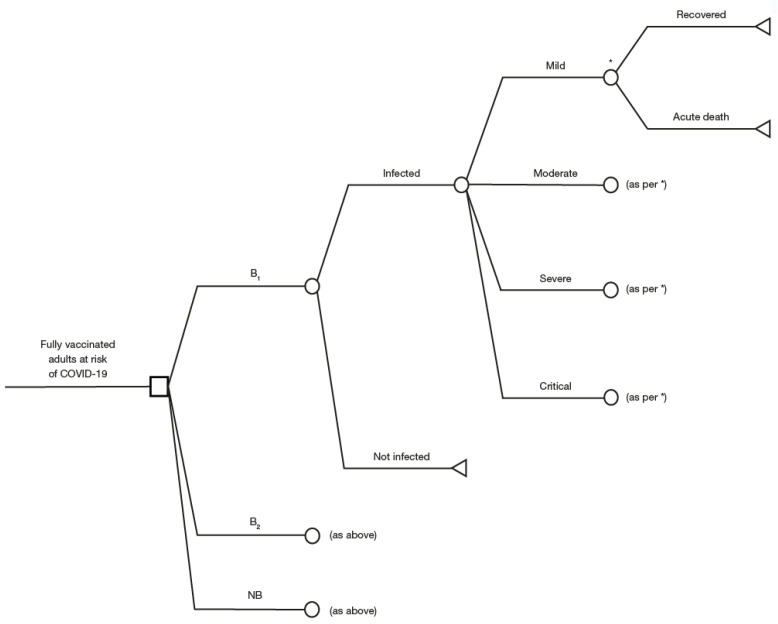
Decision tree model structure. * Recovered or acute death.

A decision tree model was developed to estimate the average per capita cost and health outcomes for the three different booster strategies. The outcomes evaluated were the number of COVID-19 cases, hospitalizations, bed-days, ICU bed-days, MV cases, and deaths, and were stratified by age group, over a 3-month time horizon for each of the booster options (B_1_, B_2_, and NB) (Figure 1).

Model design was informed from a literature review, as well as from the WHO criteria for infectious diseases model selection [45]. COVID-19 cases were divided into four categories: mild (symptomatic non-hospitalized), moderate (hospitalization requiring general ward only), severe (hospitalization requiring ICU without MV), and critical (hospitalization requiring MV), based on the WHO clinical progression scale [46].

In the decision tree model, patients could either recover or die after disease. Patients who recovered were assumed to have no long-term sequelae or mortality risk. All patients in the mild health state were assumed to recover. Long-COVID was not included because of the unavailability of data in Brazil. Boosters were not considered to lead to any severe adverse events based on the findings from clinical trials [14,47,48].

### 2.3. Methods for Analysis

Population size and vaccine coverage by age were calculated from local Brazilian data (Table S1) [49,50,51]. Derivation of the distribution of cases by severity, COVID-19 mortality, and costs are detailed in the Supplementary Material. Epidemiological and clinical inputs for the decision tree model are provided in Table S2 with MRU and treatment costs in Table S3; all costs are from 2021 and stated in US dollars.

The simplex analysis method was used to generate a BAS per age group, as all equations in the CO model were expressed as linear, including the equations of the constraints. The incremental impact in clinical results (bed-days, COVID-19 cases, hospitalization cases, ICU admissions, MV cases, and deaths) and cost results (booster cost and MRU) were defined by comparing these outputs to the control case, NB. The primary output for determining the best BAS was the number of hospital bed-days.

As different boosters are available and uncertainty still exists in their characteristics as well as the available budget, scenario analyses were performed varying the characteristics of B_2_, such as total costs, effectiveness of B_2_ for mild/moderate COVID-19, and budget constraint. Details of scenario analyses to explore uncertainty around available budgets and vaccination costs are provided in Table S4.

## 3. Results

### 3.1. Overall Analysis

**Figure 2:**
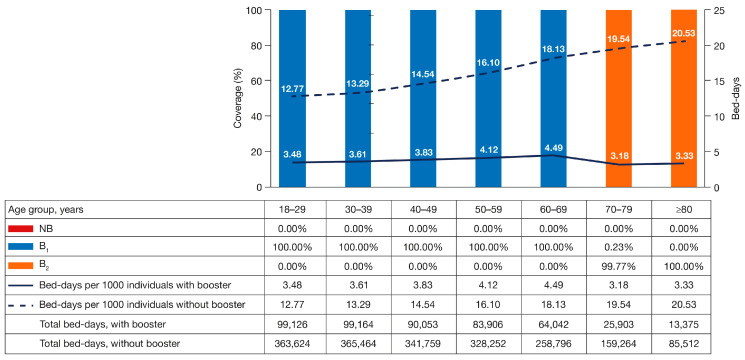
Best BAS in Brazil (base case country). BAS, booster allocation strategy.

To obtain the lowest number of bed-days, given the selected budget constraint, the analysis of the CO model recommended that adults aged <70 years be administered B_1_, and those ≥70 years be administered B_2_ in the context of the base case country, with 100% booster coverage in the target population (Figure 2).

**Figure 3:**
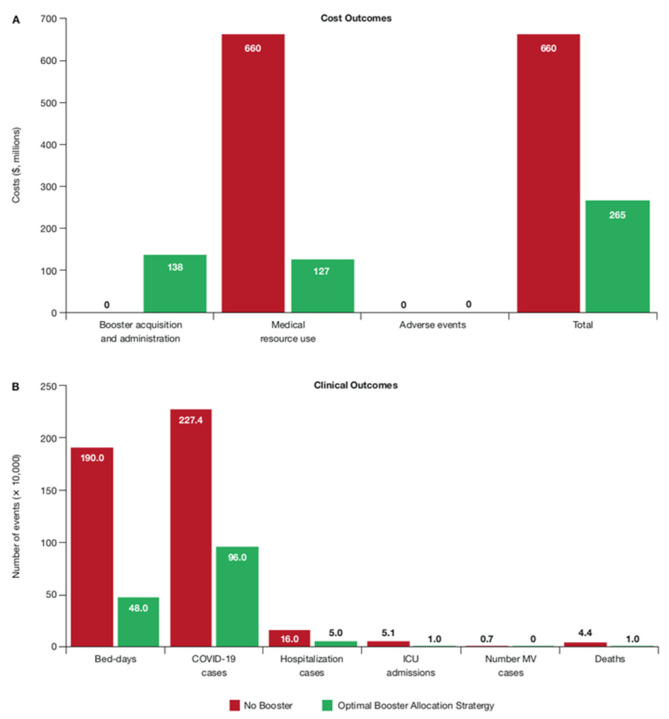
Cost (**A**) and clinical (**B**) outcomes with the Best BAS in Brazil (base-case country).

Compared with 100% NB, the best BAS resulted in reductions of 75% in bed-days (1.42 million bed-days avoided), 68% in hospitalizations (108,735 hospitalizations avoided), 90% in ICU admissions (46,185 ICU admissions avoided), 90% in MV cases (6348 MV episodes avoided), and 74% in deaths (32,549 deaths avoided). Overall, the BAS resulted in a net saving of $395 million over a 3-month duration compared with NB. This included spending $138 million on the COVID-19 booster acquisition and administration, while saving $533 million in MRU costs (MRU costs $660 million for NB vs. $127 million with the BAS) (Figure 3).

**Figure 4:**
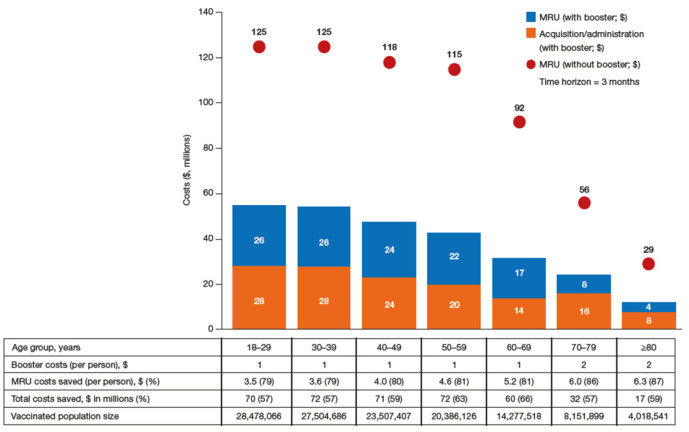
Total booster allocation strategy and no booster cost by age group. BAS, booster allocation strategy; NB, no booster; MRU, medical resource utilization.

In terms of age group-specific cost savings, the best BAS was estimated to save between $3.50 and $6.30 per capita in MRU costs, with savings increasing with age. Cumulative cost savings were highest in younger age groups, with $70–$72 million saved for every subsequent 10-year age group for those under 60 years (Figure 4 and Table S1). Age-specific cost savings are detailed in Figure 4.

### 3.2. Scenario Analysis

The best BAS (B1 + B2) was also compared with each of the single booster strategies (only B_1_ or only B_2_). Compared with B_1_ for all, which is the least expensive strategy (Table S5), the best BAS avoided an additional 20,065 bed-days and cost an additional $8 million. Using B_2_ for the entire population would avert an additional 154,602 bed-days, but add an additional $81 million to the healthcare budget, thus exceeding the $2.10 per person budget threshold. Further, a mixed booster allocation strategy (NB + B2) was also compared with BAS. It was found that it also leads to higher per person expense than the budget constraint due to high booster costs and high MRU, as compared to the best BAS identified with a combination of B1 and B2. Comparing the mixed approach of NB + B1 would generate the same results as only using B1, given that B1 is a cost saving approach with better health benefits (Table S5). Therefore, a mixed booster strategy where B_1_ and B_2_ are administered is the best approach to implement in the context of the $2.10 per person budget constraint. Other scenario analyses that varied booster effectiveness, budget constraint, and booster cost were also conducted (see Supplementary Materials).

## 4. Discussion

The findings from the CO model highlight the benefits of implementing a mixed COVID-19 booster strategy at the national level for the adult population in Brazil, the base case country. The best BAS recommended coverage of all eligible adults, with hypothetical booster B_1_ ($1) for those under 70 years of age and hypothetical booster B2 ($2) for only those 70 years and older. Compared with no booster, this strategy reduced bed-days by 75%, overall hospitalizations by 68%, and ICU admissions by 90%, leading to a 60% reduction in total costs.

Our CO model is flexible in its design, being able to assign different boosters depending on the age of the target population. While B_1_ and B_2_ were assumed equally effective against severe and critical COVID-19, B_2_ was assumed to have higher effectiveness for mild/moderate disease, while B_1_ would have a lower budget impact. Providing NB for adults was the least desirable option, with the highest costs and lowest health benefits. Comparing the best BAS with B_1_ for all, we found that BAS saved 4% more bed-days (20,065 additional bed-days) at an extra cost of $8 million. B_2_ for all provided the best clinical outcomes but, because of its higher cost, was not feasible in the base case scenario as it would overwhelm the country’s healthcare budget. B_2_ for all would avert an additional 154,602 bed-days for a net extra cost of $81 million. Hence, if the overall healthcare system can accommodate those bed-days without being overwhelmed, it may be meaningful to divert the additional $81 million to other healthcare priorities.

An advantage of using CO analysis is that it systematically and efficiently identifies the best possible solution to a problem while accounting for relevant constraints [24]. When CO models are used in a healthcare context, the results can be used immediately to advise decision makers on how certain health objectives can be achieved in accordance with a given budget. In this case, a model was developed to identify the best BAS that minimizes COVID-19–related hospital bed-days within the limits of a fixed healthcare budget. Reducing hospital bed-days was chosen as the surrogate measure of health gains due to its high importance for decision makers and payers [52,53]; hospital bed-days were used rather than ICU bed-days, as the latter would be unable to capture the overall effect of the pandemic, given that vaccinations have reduced the severity of the infections [54].

This model has been primarily designed to be flexible and can be adapted to any country/stakeholder of interest to provide guidance for determining a strategy for optimal booster dose allocation within local priorities and constraints. The objective function and constraints can be modified based on local priorities and with local data inputs. With a separate decision tree model developed to inform the optimization model, its structure allows for incorporating local disease management sets. Moreover, inputs in the decision tree can be stochastic, so they can account for a large range in the uncertainties of the results and provide results under an interesting level of variability to support decision making.

The costs included in the model are arbitrary as the exact costs are not known due to confidential discounting and local negotiations. Likewise, assumptions were made about the available health budget, as the actual budget was unknown. These values were selected to show how different budgets can lead to different vaccine allocation strategies. Not only can this model be adapted to inform the COVID-19 response in regions with different budgetary concerns, it is also an adaptable tool which can be used to respond to other non-COVID-19 epidemics and pandemics in the future.

However, we also acknowledge several limitations in the current model construct. The model accounted for only one constraint, namely budget. Other possible constraints (such as vaccine hesitancy, vaccine availability, administration limitations, and vaccine supply security) may also impact or limit booster vaccination to different extents in different countries. The model does provide flexibility to add constraints that better represent country-specific limitations. However, differences between paradigms of hospital care around the world will remain. In addition, owing to limited data availability, it has been assumed that booster effectiveness is independent of the primary vaccine administered, which may have produced inaccurate estimates of booster effectiveness. Furthermore, the parameters used to build the CO model rely on estimates of vaccine effectiveness that compare a boosted population with an unvaccinated one, whereas the comparisons generated compare boosted individuals with those who had received a primary series. This may overestimate the impact of each booster in the model. Additionally, the effectiveness of booster vaccines may be lower in response to certain (sub)variants, though the model’s flexibility can be leveraged to explore varying effectiveness against other variants. While the model considers a 3-month post-booster time horizon for the analysis, long-term benefits of implementing boosters have not been quantified. The model was created based on data from a wave of SARS-CoV-2 infection in Brazil which was predominantly composed of Gamma variant virus. The model was subsequently recalibrated to reflect parameters associated with the Omicron variant (Supplementary Information, Section 3). Though this is a limitation of the model, it does further reflect its flexibility. In addition, the model does not consider any potential impact of vaccination on infectiousness or transmission because the data on reduction in infectiousness for booster vaccines are scarce. This is a conservative assumption that also simplifies the model by not considering indirect effects. The model assumes an attack rate of 1.8% over 3 months based on the best available data. The results are very sensitive to the attack rate and at very low attack rates could result in NB being the optimal strategy. Another limitation is that only mortality due to severe COVID-19 is considered. Therefore, co-morbidities as a single cause of death are not considered. Lastly, the model only targets an adult population and does not include children and infants (>6 months) who have been approved for the boosters in some countries [55,56].

## 5. Conclusions

COVID-19 vaccine boosters can reduce both the incidence and severity of COVID-19 cases, hence reducing the burden on healthcare systems. However, healthcare decision makers face difficult decisions over which boosters to recommend, particularly because of limited budgets. This CO model represents a flexible tool that can support healthcare decision makers to identify COVID-19 vaccine booster allocation strategies that would optimize health outcomes without exceeding local budgetary constraints. The model can be adapted to any country. It can potentially be applied to any emerging disease for which there is an effective vaccine to allow decision makers to optimize the health benefits of vaccine allocation, even when healthcare resources are limited.

## Data Availability

Not applicable.

## Supplementary Materials

The following supporting information can be downloaded at: https://www.mdpi.com/article/10.3390/vaccines11020377/s1, Constrained Optimization Mathematical Model; Table S1: Population Size for Vaccinated Adult Population in Brazil; COVID-19 Case Distribution, Mortality, and Costs; Table S2: Epidemiological and Clinical Inputs for the Decision Tree Model; Table S3: MRU Duration and Cost Inputs for the Decision Tree Model; Table S4: List of Scenario Analyses; Scenario Analysis Results; Table S5: Cost-effectiveness and COVID-19 Health Outcomes of Using No Booster, Only B1 or Only B2 for All Adults. Figure S1: Scenario 4 (Budget Constraint: $2.7/Person; B_2_ Booster Cost: $2); Figure S2: Scenario 8 (Budget Constraint: $2.1/Person; B_2_ Booster Cost: $2.5); Figure S3: Scenario 12 (Budget Constraint: $2.7/Person; B_2_ Booster Cost: $2.5). References [44,46,49,50,51,57,58,59,60,61,62,63,64,65,66,67] are cited in the supplementary materials.
