# Supplementary material for: Identification of an Optimal COVID-19 Booster Allocation Strategy to Minimize Hospital Bed-Days with a Fixed Healthcare Budget
Source: Vaccines (Basel). 2023 Feb 7;11(2):377. doi: 10.3390/vaccines11020377 (PMC9965991; doi:10.3390/vaccines11020377)
Supplement: Supplementary file 1 [file vaccines-11-00377-s001.zip › vaccines-2144537-supplementary.pdf]

Supplementary Material

Identification of an Optimal COVID-19 Booster Allocation Strategy to Minimize Hospital Bed-Days with a Fixed Healthcare Budget

Ritika Kapoor [1], Baudouin Standaert [2], Edmund J. Pezalla [3], Nadia Demarteau [4], Kelly Sutton [5], Eszter Tichy [6], George Bungey [7], Sofie Arnetorp [8], Klas Bergenheim [8], Duncan Darroch-Thompson [9], Wilhelmine Meeraus [10], Lucas M. Okumura [11], Renata Tiene de Carvalho Yokota [10,12], Ray Gani [7]* and Terry Nolan [13]

This additional information has been provided by the authors to give readers additional information about their work. Supplement to Kapoor R, et al. Constrained optimization to identify the best booster allocation strategy which minimizes hospital bed-days under a fixed healthcare budget.

Table of Contents

1. Constrained Optimization Mathematical Model
2. Table S1. Population Size for Vaccinated Adult Population in Brazil
3. COVID-19 Case Distribution, Mortality, and Costs
4. Table S2. Epidemiological and Clinical Inputs for the Decision Tree Model
5. Table S3. MRU Duration and Cost Inputs for the Decision Tree Model
6. Table S4. List of Scenario Analyses
7. Scenario Analysis Results

1. Constrained Optimization Mathematical Model

The mathematical expression of the constrained optimization (CO) model is presented by an objective function with its constraints if present:

Objective to minimize $= \sum_{i=1}^{2}(B_i \ * \ X_i) + NB * X_{NB}$

Constraint = predefined healthcare budget per person of $Y_p \leq Y_{max}$

$$Y_p = \sum_{i=1}^{2}(B_i \ * \ C_i + \ B_i * \ C_{MRUi}) + NB * \ C_{MRUnb}$$

where, B_i represents the market share of different booster products (*i*=1,2, or *NB*), X_i represents the per person bed-days due to each booster option (*i*=1,2, or *NB*), NB represents no-booster, Y_p represents the average healthcare cost per person, which includes booster and MRU costs for COVID-19 disease, Y_{max} represents the maximum healthcare cost allowed per person, C_i represents the total booster cost for the different booster products (*i*=1,2), and C_{MRUi} represents the average per person medical resource use (MRU) for recovering with COVID-19 after receiving the booster (*i*=1,2, or *NB*).

2. **Table S1.** Population Size for Vaccinated (primary series) Adult Population in Brazil.

**Age Group**	**Population Size [1,2]**	**Vaccine Coverage[a] (%) [3]**	**Vaccinated Population Size (Calculated)**	**Age Group Distribution % (Calculated)**
0–5 years[a]	17,741,118	0.00	0	0.00
6–12 years[a]	17,715,603	10.00	1,771,560	1.34
13–17 years[a]	18,434,263	20.00	3,686,853	2.80
18–29 years	40,682,952	70.00	28,478,066	21.61
30–39 years	34,380,858	80.00	27,504,686	20.87
40–49 years	29,384,258	80.00	23,507,407	17.84
50–59 years	23,983,678	85.00	20,386,126	15.47
60–69 years	16,797,080	85.00	14,277,518	10.83
70–79 years	9,057,666	90.00	8,151,899	6.19
≥80 years	4,465,046	90.00	4,018,541	3.05
**Total**	**212,642,522**		**131,782,657**	**100**

[a]Vaccine coverage was estimated by calibration for the different age groups, so that the overall coverage is representative of Brazil during November 2020–April 2021 (the duration of the cross-sectional study). Overall coverage of primary series vaccination in Brazil during that time was assumed to be 62% [3]. The model only utilizes the values for age groups ≥18years.

3. COVID-19 Case Distribution, Mortality, and Costs

The total COVID-19 cases by severity were based on a local cross-sectional study in Brazil, which reported the distribution of the COVID-19 hospitalized cases to intensive care unit (ICU) admissions, non-invasive respiratory, and invasive respiratory requirements in the second wave of infections (predominantly P.1 and P.2 Gamma variants) from November 2020 to April 2021, by population age groups [4]. Using the definitions from the World Health Organization (WHO) [5], the case distribution of the hospitalized cases was categorized into moderate, severe, and critical infections. All hospitalized patients with invasive respiratory support were classified as critical, ICU admissions without invasive respiratory support were severe, and all hospitalized cases without ICU admissions were considered as moderate cases. The proportion of moderate, severe, and critical cases were recalibrated to represent the probability of severity for Omicron infection, the dominant ongoing SARS-CoV-2 variant [6]. This was done as the Omicron variant (and sub-variants) causes less severe disease than its predecessor variants and limits the benefits that can be achieved by boosters [7,8]. The relative risk of hospitalizations, ICU admission, and mechanical ventilation (MV) cases from literature were used to recalibrate the severity of COVID-19 due to Omicron [9], as compared with the Delta variant, which was dominant during the cross-sectional study. As existing severe comorbidities also significantly impact the severity of COVID-19, the case distribution was calculated separately for patients with COVID-19 with or without comorbidities using published hazard ratio for hospitalizations/death and ICU admissions/death for such patients in Australia [10]. The total mild cases were computed by subtracting the hospitalized cases from the overall reported COVID-19 cases in Brazil from November 2020 to April 2021 [11]. Mild COVID-19 were assumed to have the same distribution as moderate cases among population age groups. The case distributions are not specific to the patient vaccination status due to data unavailability.

Cases of COVID-19 mortality by age groups was estimated from the same local cross-sectional study used to determine case distribution [4]. Our model considered mortality rates to be dependent on disease severity only and did not account for the influence of existing comorbidities on the mortality risks. This assumption was taken to avoid double counting as the increased risk of developing symptomatic COVID-19 and severe/critical cases due to existing comorbidities has already been accounted for in the case distribution.

The model considers MRU costs for the treatment of COVID-19 infection that includes the cost of diagnosis, treatment, and monitoring. The MRU costs for mild infections were assumed to be equivalent to one general practitioner consultation. The cost of moderate, severe, and critical cases has been taken as reported in a local report from CONITEC, the national commission for the incorporation of technology in the health system in Brazil [12].

4. **Table S2.** Epidemiological and Clinical Inputs for the Decision Tree Model.

**COVID-19 Attack Rate**			**Value**		**Assumption**			**Source**			
**COVID-19 3-month attack rate among vaccinated population**			1.8%		An unvaccinated population is 3 times more likely to get an infection compared to the population vaccinated with a primary series [13]			Calculated based on information from ourworldindata.org [14]; aha.org [13]; worldometers.info [1]			
**Case Distribution by COVID-19 Severity Level[a]**											
			**Mild**		**Moderate**		**Severe**		**Critical**		**Source**
No Booster			92.9%		4.8%		2.0%		0.3%		Zeiser 2022 [4]
**Case Distribution by Age Groups – (Population with Comorbidities)**											
**COVID-19 Severity Level**	**0–9 Years**	**10–17 Years**	**18–29 Years**	**30–39 Years**	**40–49 Years**	**50–59 Years**	**60–64 Years**	**65–69 Years**	**70–79 Years**	**80+ Years**	**Source**
Mild[b]	94.4%	94.4%	94.4%	94.1%	94.1%	94.0%	93.9%	93.7%	93.7%	93.7%	Zeiser 2022 [4], Liu 2021 [10], Wang 2022 [9]
Moderate	4.2%	4.2%	4.2%	4.2%	4.2%	4.2%	4.2%	4.2%	4.2%	4.2%	
Severe	1.3%	1.3%	1.3%	1.5%	1.5%	1.6%	1.7%	1.8%	1.7%	1.8%	
Critical	0.2%	0.2%	0.2%	0.2%	0.2%	0.2%	0.3%	0.3%	0.4%	0.3%	
**Case Distribution by Age Groups – (Population without Comorbidities)**											
**COVID-19 Severity Level**	**0–9 Years**	**10–17 Years**	**18–29 Years**	**30–39 Years**	**40–49 Years**	**50–59 Years**	**60–64 Years**	**65–69 Years**	**70–79 Years**	**80+ Years**	**Source**
Mild[b]	91.0%	91.0%	91.0%	90.6%	90.6%	90.4%	90.2%	89.9%	89.9%	89.9%	

Moderate	6.5%	6.5%	6.5%	6.4%	6.4%	6.4%	6.4%	6.4%	6.4%	6.4%	Zeiser 2022 [4], Liu 2021 [10], Wang 2022 [9]
Severe	2.3%	2.3%	2.3%	2.7%	2.7%	2.8%	3.0%	3.1%	3.1%	3.1%	
Critical	0.3%	0.3%	0.3%	0.4%	0.4%	0.4%	0.5%	0.6%	0.6%	0.6%	
**Mortality Risks by Age Groups[c]**											
**COVID-19 Severity Level**	**0–9 Years**	**10–17 Years**	**18–29 Years**	**30–39 Years**	**40–49 Years**	**50–59 Years**	**60–64 Years**	**65–69 Years**	**70–79 Years**	**80+ Years**	**Source**
Mild	0%	0%	0%	0%	0%	0%	0%	0%	0%	0%	Zeiser 2022 [4]
Moderate	4.9%	4.9%	7.9%	7.9%	11.7%	16.9%	26.8%	38.3%	52.8%	24.6%	
Severe	4.1%	4.1%	15.2%	15.2%	21.2%	28.0%	41.6%	53.2%	64.2%	37.6%	
Critical	52.2%	52.2%	65.1%	65.1%	71.6%	78.4%	85.9%	90.9%	93.9%	83.2%	

Prevalence of Serious Comorbidities by Age Groups

**Age Group (years)**	**Value (%)**	**Source**
0–9	2	Clark 2020 [15]
10–17	3	
18–29	4	
30–39	7	
40–49	14	
50–59	23	
60–64	35	
65–69	51	
70–79	67	
80+	78	

[a]As the incidence of COVID-19 cases was not available by the vaccination status, the case distribution was calculated for the overall population and assumed to be valid for the primary series-vaccinated population. Also, the case distribution by the severity of infections was calculated for the Omicron variant, given it is the latest variant of infection and causes less severe disease than its predecessor variants.

[b]Mild cases are calculated as 1- (moderate + severe + critical disease cases).

[c]Mortality risks are assumed to be dependent on the severity of disease and not on the presence/absence of comorbidity or the variant of the infection. It is assumed that no patients die due to mild COVID-19.

5. **Table S3.** Medical Resource Use Duration and Cost Inputs for the Decision Tree Model.

**Median LOS (days) by Hospital Ward Type for COVID-19 by Severity**		
**COVID-19 Severity Level**	**Length of Stay/Bed-Days**	**Data Source**
Mild (no hospital admission)	0.00	
Moderate (Admission requires General Ward ICU/MV)	8.00	Vlachos 2021 [16]
Severe (Admission requires ICU without MV)	20.00	
Critical (Admission require Mechanical ventilation)	20.00	
**Overall MRU for Treatment of COVID-19 by Severity[a]**		
**COVID-19 Severity Levels**	**Overall MRU Costs (2021 $)**	**Source**
Mild[b]	28	Expert opinion
Moderate	1,144	CONITEC Brazil [12]
Severe[c]	9,264	
Critical[c]	9,264	

[a]MRU costs are inclusive of the cost of diagnosis, treatment, and monitoring.

[b]The MRU costs for treatment of mild infections is assumed to be similar to the cost of one GP visit.

[c]The MRU cost of severe and critical infections are a weighted average cost of both these infections.

LOS, length of stay; MRU, medical resource us; ICU, intensive care unit; MV, Mechanical ventilation.

6. **Table S4.** List of Scenario Analyses.

The total booster cost was inclusive of acquisition, administration, and logistics. Scenario analysis was conducted for B_2 cost of $2.5, to account for any additional costs due to logistics such as cold-chain storage, transportation, and administration (Scenario 2 & 3). Scenario 1 considers a higher budget constraint.

	**Boosters**	**Booster Effectiveness**		**Booster Cost (US$)**	**Budget Constraint Per Person (US$)**	**Justification**
		**Against Mild and Moderate COVID-19**	**Against Severe and Critical COVID-19**			
Base Case	B1	55%	90%	$1	$2.1	The booster that has a higher cost also has a higher effectiveness for mild and moderate cases, but both have similar effectiveness for severe infections
	B2	75%	90%	$2		
Scenario 1	B1	55%	90%	$1	$2.1	Booster effectiveness for mild and moderate infections have been varied between 60%–75% based on the range of effectiveness reported by the UK vaccine effectiveness expert panel
	B2	60%	90%	$2		
Scenario 2	B1	55%	90%	$1	$2.1	
	B2	65%	90%	$2		
Scenario 3	B1	55%	90%	$1	$2.1	
	B2	70%	90%	$2		
Scenario 4[a]	B1	55%	90%	$1	$2.7	Along with varying the effectiveness for B2, the per person budget constraint has been increased by 30% to understand the influence of increased budget
	B2	75%	90%	$2		
Scenario 5	B1	55%	90%	$1	$2.7	
	B2	60%	90%	$2		

Scenario 6	B1	55%	90%	$1	$2.7	constraint with varying booster effectiveness on the best BAS
	B2	65%	90%	$2		
Scenario 7	B1	55%	90%	$1	$2.7	
	B2	70%	90%	$2		
Scenario 8[a]	B1	55%	90%	$1	$2.1	The cost of B2 has been increased from $2 to $2.5 for all the other scenarios considered to account for any additional costs due to logistics such as cold-chain storage, transportation, and administration.
	B2	75%	90%	$2.5		
Scenario 9	B1	55%	90%	$1	$2.1	
	B2	60%	90%	$2.5		
Scenario 10	B1	55%	90%	$1	$2.1	
	B2	65%	90%	$2.5		
Scenario 11	B1	55%	90%	$1	$2.1	
	B2	70%	90%	$2.5		
Scenario 12*	B1	55%	90%	$1	$2.7	
	B2	75%	90%	$2.5		
Scenario 13	B1	55%	90%	$1	$2.7	
	B2	60%	90%	$2.5		
Scenario 14	B1	55%	90%	$1	$2.7	
	B2	65%	90%	$2.5		
Scenario 15	B1	55%	90%	$1	$2.7	
	B2	70%	90%	$2.5		

[a]Scenario explored in section 7.

7. Selected Scenario Analysis Results

Figure S1. Scenario 4 (Budget Constraint: \$2.7/Person; B_2 Booster Cost: \$2).

Budget constraint is higher than the best BAS. B2 costs the same as in the best BAS. In this scenario, because B2 is less expensive, it becomes more cost effective to vaccinate more age groups with B2.

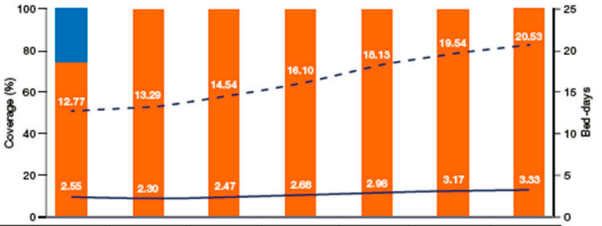

Age group, years	18–29	30–39	40–49	50–59	60–69	70–79	≥80
NB	0.00%	0.00%	0.00%	0.00%	0.00%	0.00%	0.00%
B_1	26.07%	0.00%	0.00%	0.00%	0.00%	0.00%	0.00%
B_2	73.93%	100.00%	100.00%	100.00%	100.00%	100.00%	100.00%
Bed-days per 1,000 individuals with booster	2.55	2.30	2.47	2.68	2.96	3.17	3.33
Bed-days per 1,000 individuals without booster	12.77	13.29	14.54	16.10	18.13	19.54	20.53
Total-bed days, with booster	72,610	63,383	58,123	54,717	42,235	25,873	13,375
Total-bed days, without booster	363,624	365,464	341,759	328,252	258,796	159,264	85,512

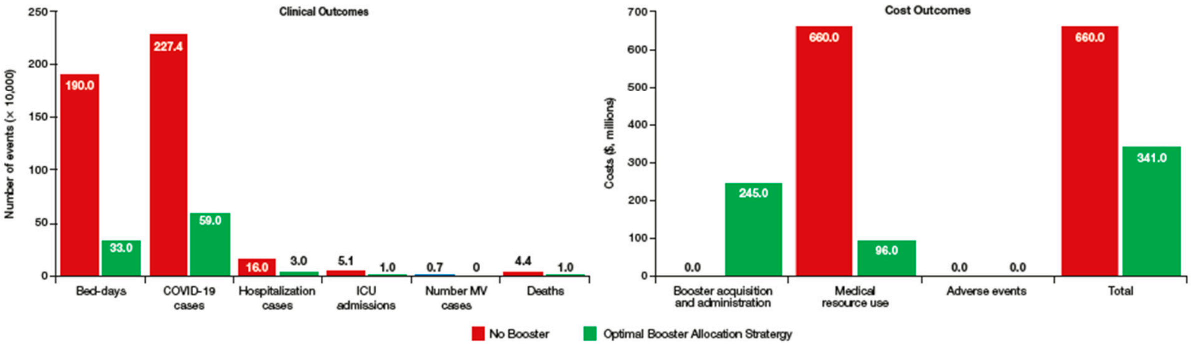

Figure S2. Scenario 8 (Budget Constraint: \$2.1/Person; B_2 Booster Cost: \$2.5).

Budget constraint is equal to the best BAS. B2 costs 25% more than in the best BAS. In this scenario, because B2 is more expensive, it is not possible to vaccinate all of the group aged 70–79 years with B2 due to budget constraints, so only part of this age group is vaccinated.

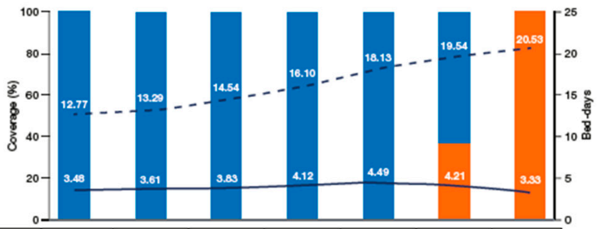

Age group, years	18–29	30–39	40–49	50–59	60–69	70–79	≥80
NB	0.00%	0.00%	0.00%	0.00%	0.00%	0.00%	0.00%
B_1	100.00%	100.00%	100.00%	100.00%	100.00%	63.72%	0.00%
B_2	0.00%	0.00%	0.00%	0.00%	0.00%	36.28%	100.00%
Bed-days per 1,000 individuals with booster	3.48	3.61	3.83	4.12	4.49	4.21	3.33
Bed-days per 1,000 individuals without booster	12.77	13.29	14.54	16.10	18.13	19.54	20.53
Total bed-days, with booster	99,126	99,164	90,053	83,906	64,042	34,324	13,375
Total bed-days, without booster	363,624	365,464	341,759	328,252	258,796	159,264	85,512

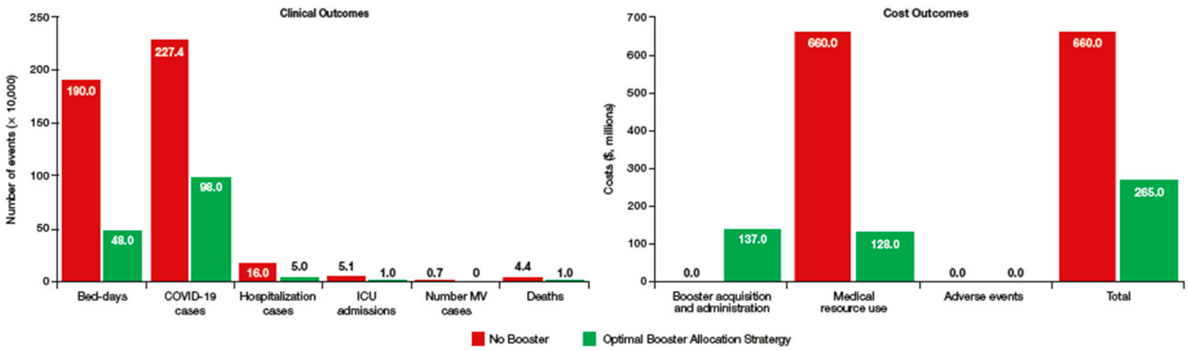

Figure S3. Scenario 12 (Budget Constraint: \$2.7/Person; B_2 Booster Cost: \$2.5)

Budget constraint is higher than the best BAS. B2 costs 25% more than the best BAS. The effect of an increased cost B2 is overcome by the increased budget constraint, and there is budget capacity to vaccinate more of the population. It is more cost effective to vaccinate those in higher age groups, allowing vaccination with B2 in all those aged 40 years and above.

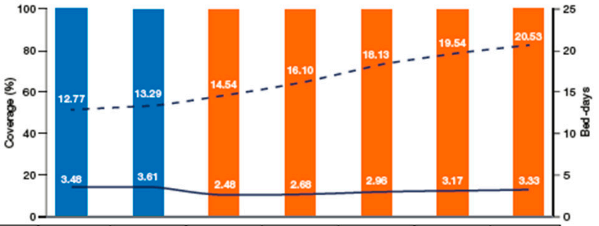

Age group, years	18–29	30–39	40–49	50–59	60–69	70–79	≥80
NB	0.00%	0.00%	0.00%	0.00%	0.00%	0.00%	0.00%
B_1	100.00%	100.00%	0.64%	0.00%	0.00%	0.00%	0.00%
B_2	0.00%	0.00%	99.36%	100.00%	100.00%	100.00%	100.00%
Bed-days per 1,000 individuals with booster	3.48	3.61	2.48	2.68	2.96	3.17	3.33
Bed-days per 1,000 individuals without booster	12.77	13.29	14.54	16.10	18.13	19.54	20.53
Total bed-days, with booster	99,126	99,164	58,327	54,717	42,235	25,873	13,375
Total bed-days, without booster	363,624	365,464	341,759	328,252	258,796	159,264	85512

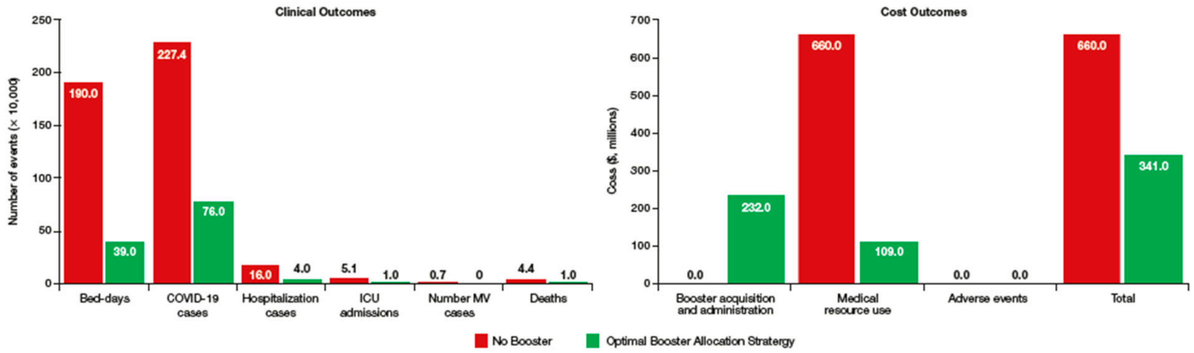

Table S5. Cost-effectiveness and COVID-19 Health Outcomes of Using No Booster, Only B1 or Only B2 for All Adults

	Total Costs per individual	Incremental Costs (compared with B1)	Expected COVID-19 Cases over 3 months	Expected COVID-19 Deaths over 3 months	Mild COVID-19 Cases	Hospitalizations (not including ICU admissions and MV cases)	ICU Admissions	MV Cases	Bed-Days
No Booster	$ 5.23	-$ 3.19	0.0180	0.0003	0.0167	0.0013	0.0004	0.0001	0.0150
Booster 1	$ 2.04	-	0.0080	0.0001	0.0075	0.000430	0.0000	0.0000	0.0039
Booster 2	$ 2.74	-$ 0.71	0.0044	0.0001	0.0042	0.000257	0.0000	0.0000	0.0025

ICU = intensive care unit; MV = mechanical ventilation

References

1. Worldometer. Brazil population (LIVE). Available online: https://www.worldometers.info/world-population/brazil-population/ (accessed on 17 June 2022).
2. Instituto Brasileiro de Geografia e Estatística. Projeção da população do Brasil e das Unidades da Federação. Available online: https://www.ibge.gov.br/apps/populacao/projecao/ (accessed on 17 June 2022).
3. Institute for Health Metrics and Evaluation. COVID-19 results briefing. Brazil. Available online: https://www.healthdata.org/sites/default/files/files/135_briefing_Brazil_1.pdf (accessed on 17 June 2022).
4. Zeiser, F.A.; Donida, B.; da Costa, C.A.; Ramos, G.O.; Scherer, J.N.; Barcellos, N.T.; Alegretti, A.P.; Ikeda, M.L.R.; Muller, A.; Bohn, H.C.; et al. First and second COVID-19 waves in Brazil: a cross-sectional study of patients' characteristics related to hospitalization and in-hospital mortality. *Lancet Reg Health Am* **2022**, *6*, 100107. doi:10.1016/j.lana.2021.100107.
5. WHO Working Group on the Clinical Characterisation and Management of COVID-19 infection. A minimal common outcome measure set for COVID-19 clinical research. *Lancet Infect Dis* **2020**, *20*, e192-e197. doi:10.1016/S1473-3099(20)30483-7.
6. UK Health Security Agency. COVID-19 vaccine surveillance report. Week 16. Available online: https://assets.publishing.service.gov.uk/government/uploads/system/uploads/attachment_data/file/1070356/Vaccine-surveillance-report-week-16.pdf (accessed on 17 June 2022).
7. Christensen, P.A.; Olsen, R.J.; Long, S.W.; Snehal, R.; Davis, J.J.; Ojeda Saavedra, M.; Reppond, K.; Shyer, M.N.; Cambric, J.; Gadd, R.; et al. Signals of significantly increased vaccine breakthrough, decreased hospitalization rates, and less severe disease in patients with coronavirus disease 2019 caused by the omicron variant of severe acute respiratory syndrome coronavirus 2 in Houston, Texas. *Am J Pathol* **2022**, *192*, 642-652. doi:10.1016/j.ajpath.2022.01.007.
8. Iuliano, A.D.; Brunkard, J.M.; Boehmer, T.K.; Peterson, E.; Adjei, S.; Binder, A.M.; Cobb, S.; Graff, P.; Hidalgo, P.; Panaggio, M.J.; et al. Trends in disease severity and health care utilization during the early omicron variant period compared with previous SARS-CoV-2 high transmission periods - United States, December 2020-January 2022. *MMWR Morb Mortal Wkly Rep* **2022**, *71*, 146-152. doi:10.15585/mmwr.mm7104e4.
9. Wang, L.; Berger, N.A.; Kaelber, D.C.; Davis, P.B.; Volkow, N.D.; Xu, R. Comparison of outcomes from COVID infection in pediatric and adult patients before and after the emergence of Omicron. *medRxiv* **2022**. doi:10.1101/2021.12.30.21268495.
10. Liu, B.; Spokes, P.; He, W.; Kaldor, J. High risk groups for severe COVID-19 in a whole of population cohort in Australia. *BMC Infect Dis* **2021**, *21*, 685. doi:10.1186/s12879-021-06378-z.
11. Mathieu E; Ritchie, H.; Rodés-Guirao, L.; Appel, C.; Giattino, C.; Ortiz-Ospina, E.; Hasell, J.; Macdonald, B.; Dattani, S.; Roser, M. Brazil: coronavirus pandemic country profile. Available online: https://ourworldindata.org/coronavirus/country/brazil (accessed on 17 June 2022).

12. Ministério da Saúde. Casirivimabe/imdevimabe para pacientes de alto risco infectados por SARS-CoV-2. Available online: http://conitec.gov.br/images/Relatorios/2021/20210712_Relatorio_635_Regn-Cov2_COVID-19_P1537.pdf (accessed on 17 June 2022).
13. American Hospital Association. CDC: Unvaccinated adults 3-5 times more likely to get Omicron infection. Available online: https://www.aha.org/news/headline/2022-01-21-cdc-unvaccinated-adults-3-5-times-more-likely-get-omicron-infection (accessed on 9 November 2022).
14. Mathieu, E.; Ritchie, A.J.; Rodés-Guirao, L.; Appel, C.; Giattino, C.; Hasell, J.; Macdonald, B.; dattani, S.; Beltekiam, D.; Ortiz-Ospina, E.; et al. Coronavirus pandemic (COVID-19). Available online: https://ourworldindata.org/coronavirus (accessed on 2 November 2020).
15. Clark, A.; Jit, M.; Warren-Gash, C.; Guthrie, B.; Wang, H.H.; Mercer, S.W.; Sanderson, C.; McKee, M.; Troeger, C.; Ong, K.I.; et al. How many are at increased risk of severe COVID-19 disease? Rapid global, regional and national estimates for 2020. *medRxiv* **2020**, 2020.2004.2018.20064774. doi:10.1101/2020.04.18.20064774.
16. Vlachos, S.; Wong, A.; Metaxa, V.; Canestrini, S.; Lopez Soto, C.; Periselneris, J.; Lee, K.; Patrick, T.; Stovin, C.; Abernethy, K.; et al. Hospital mortality and resource implications of hospitalisation with COVID-19 in London, UK: a prospective cohort study. *Crit Care Res Pract* **2021**, *2021*, 8832660. doi:10.1155/2021/8832660.
